# Cystatin antibodies interfere with ovary development in *Haemaphysalis doenitzi* (Acari: Ixodidae)

**DOI:** 10.1371/journal.pntd.0013064

**Published:** 2025-05-07

**Authors:** Songbo Zhang, Zhihua Gao, Ahmed H. Ghonaim, Weijia Xing, Weikang Zhao, Jiayi Zhang, Xiaolong Yang

**Affiliations:** 1 Hebei Key Laboratory of Animal Physiology, Biochemistry and Molecular Biology, Hebei Collaborative Innovation Center for Eco-Environment, Ministry of Education Key Laboratory of Molecular and Cellular Biology, College of Life Sciences, Hebei Normal University, Shijiazhuang, China; 2 National Key Laboratory of Agricultural Microbiology, College of Veterinary Medicine, Huazhong Agricultural University, Wuhan, China; 3 Desert Research Center, Cairo, Egypt; Creighton University, UNITED STATES OF AMERICA

## Abstract

Anti-tick vaccines are gaining attention as a strategy to prevent tick infestations by activating the immune response of the host. Antibodies produced by the host inhibit tick growth and reproduction, but the molecular mechanism remains to be clarified. In this study, we investigated the effects of cystatin antibodies on the ovarian function of *Haemaphysalis doenitzi*. Histological analysis revealed that exposure to cystatin antibodies resulted in a significant reduction in the number of eggs produced and caused severe damage to the ovarian tissue structure. Immunofluorescence experiments confirmed the significant expression of cystatin within the ovary. Proteomics and phosphoproteomics identified 31 and 10 differentially expressed proteins in the relevant pathways, respectively. These changes in protein levels were found to be regulated by various mechanisms, including ribosomes activity, regulation of actin cytoskeleton, RNA transport, the TCA cycle, drug metabolism, and mTOR signaling pathways. Notably, there was high expression of tropomyosin and low expression of glutathione S-transferase (GST) during ovarian detoxification. Enzyme activity assays indicated a significant down-regulation of GST enzyme activity in the immunized group, suggesting that cystatin antibodies impaired the detoxification capacity of the ticks. Both tropomyosin and GST were successfully cloned and designated as HD-TPMa and HD-GSTa, respectively. RNA interference (RNAi) successfully knocked down the target gene. Ticks subjected to immersion in cystatin antibodies exhibited a significantly increased mortality rate after 72 hours. This study elucidated the molecular mechanism by which cystatin antibodies inhibit the growth and development of tick ovaries, providing an important scientific basis for the development of effective tick ovary control strategies.

## Summary

The *Haemaphysalis doenitzi* is an important vector tick harvested from the body surface of sheep in Cangxi County, Sichuan Province, China, and is capable of transmitting a variety of zoonotic diseases. In recent years, its distribution has been expanding and it has been found in many parts of China, which further exacerbates public and animal health risks. Previous studies have found that protein vaccines show great potential for tick control and that cystatin protein vaccines are effective against ticks of the same genus. This vaccine effectively protects livestock from ticks, which could improve the safety of meat products and reduce the potential risk of tick-borne diseases. We systematically explored the mechanism of action of cystatin antibodies on the ovaries of *H. doenitzi* and found that cystatin antibodies significantly reduced glutathione S-transferase (GST) activity and impaired the detoxification capacity of the tick. Understanding the mechanisms of tick ovarian development is critical to understanding the ecological characteristics of ticks and the patterns of tick-borne disease transmission.

## Introduction

Ticks are notorious for their specialized blood-sucking habits and high reproductive capacity, posing serious threats to human and animal health as vectors of various disease pathogens [1]. *Haemaphysalis doenitzi* is an ectoparasitic tick that often parasitizes birds and small mammals and can transmit diseases such as babesiosis, posing a threat to animal and human health [2]. In recent years, with the increase in the number of pets, the incidence of tick-borne zoonoses in pets has increased, threatening public health safety [3]. *H. doenitzi* is found in regions including Australia, New Zealand, Korea, Japan, China, and parts of Southeast Asia [4]. On Antikythira Island, Greece, this tick species infests the valuable *Falco eleonorae* population [5]. In China, particularly on Chongming Island, ticks pose a potential risk to local ecosystems and human health [6]. Additionally, viruses circulate between birds and ticks, complicating disease transmission as pathogens spread to new areas through bird migration [7]. Therefore, implementing control and preventive measures for *H. doenitzi* is essential for safeguarding human and animal health.

Current tick control strategies primarily involve chemical methods, vaccination, and physical barriers. However, the overuse of chemicals has led to increased tick resistance and environmental pollution. This has prompted the exploration of essential oils and anti-tick vaccines derived from plant extracts, which are beneficial to humans but lethal to ticks. For instance, essential oils from *Callistemon viminalis* and *Satureja montana* L. have demonstrated significant toxic effects on the ovaries of *Rhipicephalus microplus*, impairing oocyte development and reducing reproductive capabilities [8,9]. The molecular mechanisms through which anti-tick vaccines confer protection against ticks remain largely unexplored. Multiple molecules in tick ovaries, including anti-inflammatory, anticoagulant, and immune response factors, play vital roles in detoxifying external toxins. Cystatins, as natural inhibitors of cysteine proteases, play a key role in the immune response of insects, aiding ticks in regulating immune cell activity and inflammatory responses [10]. Previous studies have demonstrated that recombinant vaccination with cystatin proteins prompts the host to produce antibodies, significantly decreasing tick oviposition and hatchability. Four family 2 cystatin antibodies were selected for our study. Despite their different expression levels and mechanisms of action in tick saliva, rHDcyst-1 and rHDcyst-2 mainly inhibit host immune responses, while rHDcyst-3 and rHDcyst-4 primarily regulate tick physiological processes. However, they share a common role in the immune mechanism of ticks, with significant control effects on ticks at 64.1%, 51.8%, 55.9%, and 63.2% respectively [1,2]. These findings provide new perspectives into tick defense and control of reproduction and development through the modulation of detoxification mechanisms in tick ovaries.

In this study, we investigated the molecular mechanisms by which cystatin antibodies affect tick growth and development, particularly focusing on detoxification functions. In this study, we explored the molecular mechanisms underlying the effects of cystatin antibodies rHDcyst-1, rHDcyst-2, rHDcyst-3, and rHDcyst-4 on tick growth and development, with a particular focus on their detoxification functions. The ovary, the site of egg production for female ticks, plays a crucial role in maintaining normal developmental processes in the presence of external toxins through a complex detoxification mechanism [11]. We examined the morphology of the toxin-infected ovaries and utilized proteomics and phosphoproteomics techniques to uncover the molecular dynamics in response to toxins. In addition, RNA interference (RNAi) was employed to knock down target genes, and immersion tests were conducted on *H. doenitzi* using cystatin antibodies derived from New Zealand white rabbits to assess the role of functional molecules in the ovary in resisting toxin exposure. This study underscores the regulatory role of ovarian molecules in tick susceptibility to cystatin antibodies, aiding in reducing tick numbers and tick-borne diseases, thus enhancing public health security.

## Materials and methods

### Ethics Statement

All animal experiments involving ticks and New Zealand Greater White Rabbit were approved by the Animal Ethics Committee of Hebei Normal University (Approval No. 2023LLSC040).

### Ticks

Specimens of *Haemaphysalis doenitzi* were collected from the surface of the ears of sheep in Cangxi County, Sichuan, China. The ticks were reared in an artificial climate incubator at 26°C and 75% relative humidity with a 16: 8 h of light: dark cycle. During the life transmission process, ticks were placed on cloth ear covers positioned on the ears of New Zealand white rabbits to facilitate reproduction through blood-sucking.

### Ovary dissection and section analysis

To study the response of tick ovaries to rabbit-derived cystatin antibodies, the following experimental design was implemented: the control group received 500 μL of PBS combined with 500 μL of Freund’s adjuvant, while the experimental group received 500 μL of recombinant HD cystatin proteins (rHDcyst-1, rHDcyst-2, rHDcyst-3, and rHDcyst-4) combined with 500 μL of Freund’s adjuvant. Six four-month-old New Zealand Large White rabbits (three males and three females) were used in each group. The immunization was carried out subcutaneously on the backs of the rabbits in a multiplexed manner across three sessions. The antibody concentration in the rabbit serum following immunization was approximately 6 mg/ml. Each rabbit received three immunizations at the following time points: the first immunization on Day 0, the second on Day 14, and the third on Day 28. The engorged ticks were dissected five days after detachment from the rabbit’s ears. Complete ovaries from 25 adult female ticks were dissected and placed individually into EP tubes filled with tissue fixative. Subsequent sectioning was performed, followed by staining procedures. Hematoxylin and eosin (HE) was utilized to visualize ovarian morphology, with HE stains resulting in blue nuclei and red cytoplasm. For immunohistochemistry, HDcyst-1, HDcyst-2, HDcyst-3, and HDcyst-4 antibodies served as primary antibodies, while HPR-labeled goat anti-rabbit IgG (H + L) acted as a secondary antibody. This process stained the nuclei of the cells blue and the antigen-antibody binding sites brownish-yellow. Immunofluorescence localization experiments were carried out using DAPI for nuclear staining, producing blue colors, and CY3 fluorescein for visualizing antigen-antibody binding sites, which appeared red. Photographs of these sections were captured using a ZEISS microscope (Zoom.V16).

### Trypsin digestion

In the proteomics and phosphoproteomics studies, the experiment was meticulously designed with five treatment groups, each comprising n = 5 independent biological samples, with three replicates per group. We homogenized the ovarian samples using a tissue homogenizer in lysis buffer containing protease and phosphatase inhibitors. After homogenization, the samples were centrifuged to remove debris, and the supernatant was collected for protein isolation. To dissolve the proteins, 20 μL of water was added, maintaining a protein-to-water ratio of 5:1. Following this, 1 μL of DTT was introduced, and the mixture was incubated at 37°C for 1 h. Subsequently, 2 μL of IAA was added, and the solution was left at 26°C for 30 min in the dark. For digestion, 2 μg of trypsin (USA Promega, 1: 50 W/W) were used, and 23 μL of 100 mM NH_4_HCO_3_ was added to the trypsin. The enzymatic reaction was carried out in a water bath at 37°C overnight. To purify the digested peptides, a C18 solid phase extraction column (desalted) was activated with 1 mL of 100% acetonitrile (ACN, Anpel, China). The column was then washed three times with 1 mL of mass spectrometry-grade water. The enzyme solution was centrifuged at 12,000 × g for 5 min, and the supernatant was transferred to a new centrifuge tube. The protein solution was repeatedly pumped through a glass needle once, and the extraction column was washed three additional times with 1 mL of mass spectrometry water. Peptide gradients were eluted into new Ep tubes using 30% and 40% ACN. The concentrations of the purified peptide concentrations were normalized to expected values using a BCA protein assay kit (Pierce, Rockford, IL, USA). Digestion efficiency was monitored using liquid chromatography-mass spectrometry (HPLC-MS; Orbitrap Exploris 480, ThermoFisher Scientific, USA).

### Phosphorylated peptide enrichment

For the enrichment of phosphorylated peptide, Phoss-TiO₂ Beads (GL Sciences Inc, Japan) were first activated. To do this, 8 mL of ethanoic acid solution was added to a reaction tube along with 0.5 mg of beads and mixed thoroughly. The beads were then centrifuged at 8200 × g for 1 min, the supernatant was discarded. This resuspension process was repeated twice, after which 8 mg of beads were resuspended in 0.8 mL of ethanoic acid solution. Next, 500 μL of ethanoic acid solution was added to the spin-dried ovarian protein and mixed well. The resuspended ovarian protein was then added to the beads and incubated for 1.5 h at room temperature. After incubation, the supernatant (non-phosphorylated protein) was centrifuged at 8200 × g for 1 min and transferred to a new tube for storage. The beads were subsequently washed by adding 500 μL of glycolic acid solution to completely suspend them. The beads were transferred to new tubes and centrifuged at 8200 × g for 1 min, discarding the supernatant. This washing step was repeated with 500 μL of 50% acetonitrile solution, followed by another centrifugation at 8200 × g for 1 minute, discarding the supernatant. Finally, 500 μL of 20 mM ammonium acetate solution was added to suspend the beads, centrifuged at 8200 × g for 1 min, and the supernatant was discarded. For the final elution, 100 μL of 5% NH₄OH solution was added and incubated at room temperature for 5 min, then centrifuged at 8200 × g for 1 min. The supernatant was transferred to a new tube, and this step was repeated twice. The final supernatant was then spun-dried and prepared for sampling.

### DIA quantitative proteomic analysis

In this study, quantitative analyses of ovaries from various immunological treatments were performed to identify phosphorylated proteins enriched in the phosphopeptide fraction. The experiments utilized the High-Select TiO_2_ phosphopeptide enrichment kit (Thermo Fisher Scientific, USA) following the manufacturer’s guidelines. The enriched phosphopeptides were subsequently lyophilized, and this procedure was repeated three times across 15 ovarian samples. Samples were initially processed in a solution consisting of 99.9% water and 0.1% trifluoroacetic acid (FA). For DIA quantification, a combination of LC-MS and UHPLC systems (Vanquish Neo, GER) was employed, coupled with an Orbitrap Exploris 480 mass spectrometer (Thermo Fisher, USA). Each ovarian protein was loaded onto a C18 RP trap column with a 5 μm particle size (300μm ID × 5mm L, PEPMAPNEO; Thermo, USA). A C18 RP analytical column (1.5 μm particle size, 75 μm ID × 250 mm length; Waters, USA) was used with a flow rate of 300 nL/min. The ovarian protein was ionized in the Orbitrap Exploris 480 mass spectrometer (voltage: 2.3 KV, heating capillary temperature: 350˚C). The raw data on protein identification were deposited on the Proteome Exchange Consortium platform (http://proteomecentral.proteomexchange.org) with the data storage ID PXD055423.

### Bioinformatic analyses

Bioinformatics analysis was performed to screen tick ovaries for proteins associated with cystatin antibodies. Cluster analysis of proteins was conducted using the GProX tool, which identifies groups of proteins with similar expression patterns. Four clusters were established for this analysis, with expression change thresholds set at 0.58 for up-regulation and –0.58 for down-regulation, corresponding to 1.5 and 0.67 times the change rate of the original data. For further correlation analysis, partial least squares identification maps, rain cloud maps, thermograms, volcano maps, and KEGG and GO database queries(https://pantherdb.org/), the Wukong Cloud platform was utilized (https://www.omicsolution.com/wkomics/main/) This comprehensive approach aimed to elucidate the biological functions and molecular pathways associated with these proteins.

### Enzyme activity

The glutathione S-transferase activity was assessed using a kit (Solarbio, CHINA). Ovarian homogenization was performed in an ice bath at a ratio of 1:5–10 (tissue mass in grams to reagent volume in milliliters). For example, approximately 0.1 g of ovarian was mixed with 1 mL of Reagent 1. We dissected ovaries from engorged female ticks and homogenized the samples in lysis buffer containing protease and phosphatase inhibitors to ensure protein stability. The mixture was then centrifuged at 8000 × g for 10 min at 4°C, and the supernatant on ice was retained for measurement. Before the assay, the enzymatic instrument was warmed up for more than 30 min, and the wavelength was set to 340 nm. The spectrophotometer was zeroed with distilled water. A portion of Reagent II was preheated at 37°C for 15 min, and then ovarian proteins, along with reagents II and III, were added to a 96-well UV plate and mixed quickly. The absorbance was recorded at 340 nm at two time points: 10 seconds after mixing (A1 measurement) and after a 5-minute incubation at 37°C (A2 measurement). The following formula was used to calculate the change in absorbance (ΔA): ΔA = (A2 measurement - A1 measurement) - (A2 blank - A1 blank). Blank tubes were prepared 1–2 times for calibration.

### RNA interference

Primers for tropomyosin and glutathione S-transferase were designed using Primer 5.0 software, and sequencing analyses were conducted to ensure their specificity and validity (Table 1). Using the SWISS-MODEL platform (https://swissmodel.expasy.org/), we predicted the three-dimensional structure of the two proteins. Cross-species phylogenetic analysis was performed using the neighbor-joining method implemented in MEGA 7.0 to construct the phylogenetic tree based on protein sequences. Total RNA was extracted from ticks using the RNA extraction kit (AxyPrep Multisource Total RNA, China) following standard operating procedures. *In vitro* transcription was performed to synthesize RNA. Amplification of target genes were achieved through PCR, and products were purified using the EasyPure Rapid Gel Extraction Kit for subsequent synthesis of double-stranded RNA (dsRNA). The injection was performed using a microinjector to ensure that the dsRNA could enter the cells, with 1000 ng of dsRNA being injected into unfed female ticks. To assess the effectiveness of RNAi, SYBR Green chemistry was used for qPCR assays. Subsequently, a cystatin antibody immersion test was carried out. Ticks were treated with the antibody at a concentration of 6 mg/mL for 5 min, after which the ticks were dried on filter paper. The survival of the ticks was monitored after 72 h to assess the toxic effects of the antibody. Statistical analyses were performed using the Student’s t-test and Tukey’s Honest Multiple Comparison Test.

**Table 1:** Primers for polymerase chain reaction (PCR).

Classification	Gene	Forward primer (5 ′→ 3)	Reverse primer (5 ′→ 3)
Cloned ORF	HD-TPMa	ATGTCTTCCGGCGTGACG	CTACTTGCGCGACGATTG
	HD-GSTa	ATGGCCGTGGAGCTGTACA	TCACTTGGGGGGGCC
RNA Interference	HD-TPMa	AACGAGGCCAAGACGGTG	TCGAAGACGCAGTAGCGG
	HD-GSTa	GGTTCAAGGGCCAAGAGC	GCAGCTGCGGGAACTTCT
	GFP	GACGTAAACGGCCACAAGT	GCTTCTCGTTGGGGTCTTT
Quantitative real-time PCR	HD-TPMa	TCGAAGACGCAGTAGCGG	TCGCACGCCTGGACATAC
	HD-GSTa	GGCCGTGGAGCTGTACAA	TGATGGTCGGCACCGTAT
	β-Actin	CGTTCCTGGGTATGGAATCG	TCCACGTCGCACTTCATGAT

Note：RNA interference primer design with T7 promoter and protective bases: GGATCCTAATACGACTCACTATAGGG

## Results

### Ovary histology

**Fig 1:**
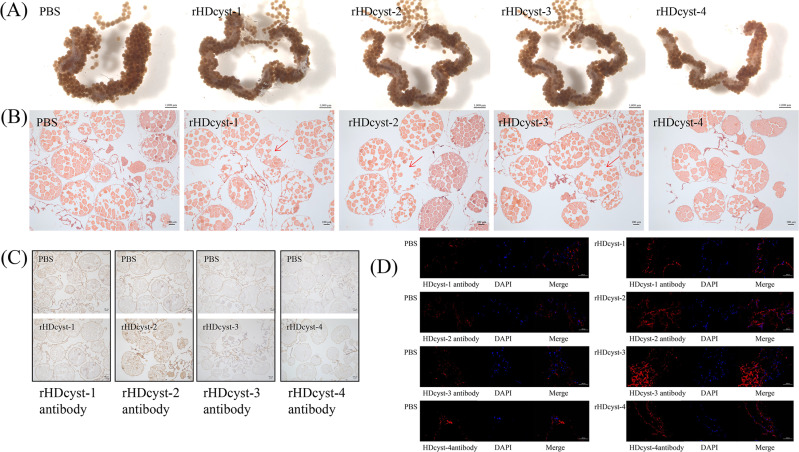
The ovary of ticks infected with cystatin antibodies. (A) Comparison of ovarian morphology. (B) Ovarian HE staining. Arrows to indicate fractures. (C) Ovarian immunohistochemistry. (D) Ovarian immunofluorescence staining. The experimental groups included PBS as the control, and the treatment groups consisted of rHDcyst-1 antibody, rHDcyst-2 antibody, rHDcyst-3 antibody, and rHDcyst-4 antibody.

Ticks of uniform size were dissected to observe the morphological structure of the ovaries under a microscope, using a scale bar of 1000 μm. In the PBS group, the ovaries appeared structurally normal and intact, characterized by numerous, well-formed, and closely arranged eggs. In contrast, the rHDcyst-4 group exhibited smaller ovaries with fewer, sparsely arranged eggs (Fig 1A). HE staining revealed that the ovaries of the PBS group contained more mature oocytes with complete cellular structures and normal development. However, in the rHDcyst-1, rHDcyst-2, and rHDcyst-3 groups, some cellular structures were observed to be ruptured; rHDcyst-4 was excepted (Fig 1B). For immunohistochemistry, sections were incubated with antibodies. In the PBS group, antibody binding sites were widely distributed, mainly located on the cell membranes of mature oocytes. The rHDcyst-1 and rHDcyst-2 groups exhibited a relatively higher number of antibody binding sites, while the rHDcyst-3 and rHDcyst-4 groups had fewer antibody binding sites, primarily concentrated in immature oocytes, appearing lighter in color (Fig 1C). Immunofluorescence staining assay was performed using DAPI to label nuclei (blue) and cystatin antibody to detect recombinant proteins (red). In the PBS control group, the red fluorescent signal was weak. Conversely, the rHDcyst-1 to rHDcyst-4 immunized groups showed significantly enhanced red fluorescent signals, indicating the expression and localization of recombinant proteins within the cells (Fig 1D). Using Fiji software, we performed quantitative analysis of the fluorescence intensity of ovarian-specific antibodies. Statistical analysis via t-test revealed that the experimental group exhibited significantly higher mean fluorescence intensity compared to the control group (S1 Fig) (*P* < 0.05). This suggests that cystatin plays a key role in tick ovarian immunomodulation, with different treatments potentially affecting tick reproduction by altering ovarian morphology.

### Protein identification

**Fig 2:**
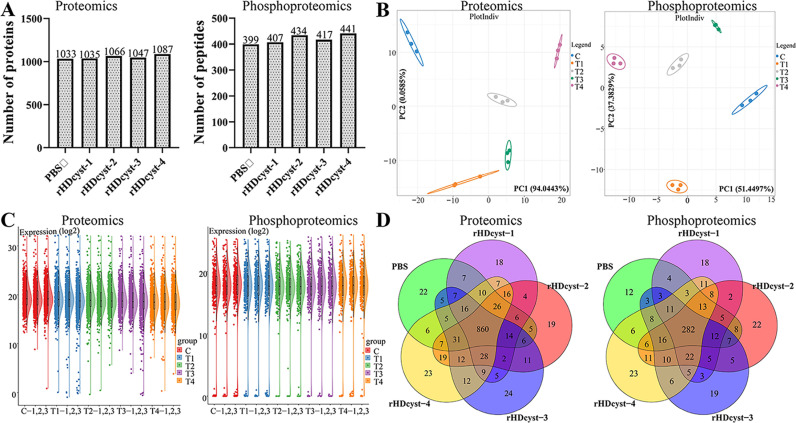
(A) Identification of the quantity of ovaries and four treatment antigens of female ticks after removing missing values. (B) The Partial least squares-discriminant analysis of the ovaries of female ticks treated with different antigens. (C) Raincloud plot, which is a combination of violin plot, boxplot, and scatter plots, shows the same median level between the treatment groups in different tissues. Cloud: data distribution; Umbrella: a tiled box diagram; Rain: original data dot matrix, with the middle part being the box diagram. (D) Venn diagram showing the overlap and specificity of protein identification between different treatment groups. Under five different treatments, 860 intersection proteins were identified in the ovary of *Haemaphysalis doenitzi*, and 282 intersection peptides were identified in the ovary. C: PBS control group; T1: rHDcyst-1 treatment group; T2: rHDcyst-2 treatment group; T3: rHDcyst-3 treatment group; T4: rHDcyst-4 treatment group; All data showed satisfactory reproducibility.

**Fig 3:**
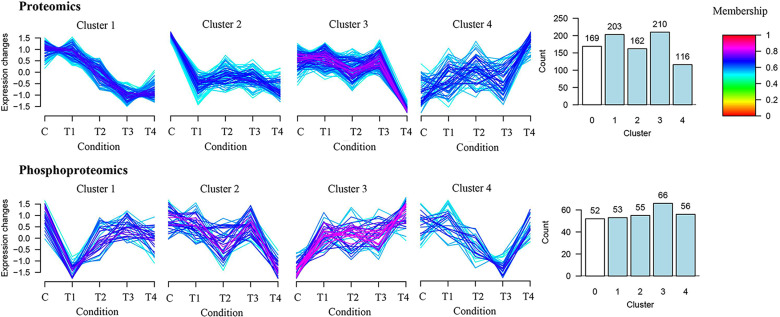
Cluster analysis of intersected proteins and peptides of the ovary treated with different antigens. The intersected proteins and peptides were divided into four categories. 0 on the x-axis of the cluster distribution represents the number that has not changed significantly; 1 represents the number classified into Cluster 1; 2 represents the number classified into Cluster 2; 3 represents the number classified into Cluster 3; and 4 represents the number classified into Cluster 4. C: PBS control group; T1: rHDcyst-1 treatment group; T2: rHDcyst-2 treatment group; T3: rHDcyst-3 treatment group; T4: rHDcyst-4 treatment group; All data showed satisfactory reproducibility.

**Fig 4:**
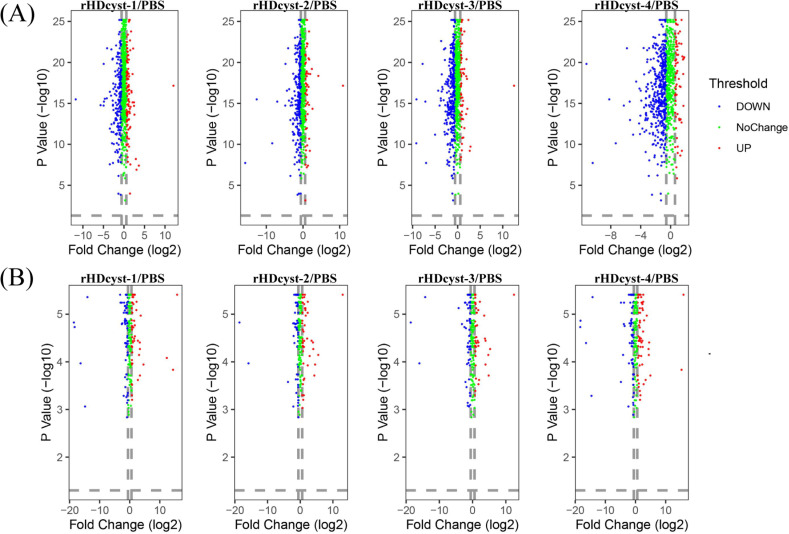
The volcano plot shows the quantitative changes of differentially expressed proteins in the ovary of female ticks exposed to cystatin antibodies. The red triangle represents up-regulation, the blue square down-regulation, and the green circle proteins with unchanged expression. (A) Proteomics; (B) Phosphoproteomics.

Using DIA quantitative proteomics technology, we detected 1033, 1035, 1066, 1047, and 1087 proteins in the control and experimental ovaries. Phosphoproteomics techniques identified 399, 407, 434, 417, and 441 phosphorylated polypeptide segments in the ovaries (Fig 2A). To ensure the accuracy of the experimental results, PLS-DA and correlation analyses were performed on the identified proteins across each group, demonstrating a high degree of reproducibility among the three replicate experiments (Fig 2B). A heatmap visualized the expression level changes of common proteins and phosphorylation-modified polypeptides between different groups, revealing small errors (Fig 2C). Venn diagrams illustrated the identification results of ovarian proteins under different treatments, highlighting the overlap and specificity of protein identification between groups (Fig 2D). A total of 860 intersecting proteins were identified in DIA quantitative proteomics, along with 282 intersecting phosphorylated peptide segments in phosphoproteomics. The expression of these crossover proteins was analyzed through clustering, identifying expression proteins as those with more than a 1.50-fold or less than a 0.67-fold change in expression (Fig 3). The GProX platform categorized protein expression trends in each treatment, including continuous up-regulation, down-regulation, and irregular changes. This analysis aided in identifying dynamic changes in protein expression under cystatin antibody toxin stress. Volcano map analysis results demonstrated the variations in the expression levels of ovarian proteins and phosphorylation-modified peptides (Fig 4). In the different cystatin antibody treatment groups, we observed a higher number of down-regulated proteins and fewer up-regulated proteins in the ovaries (Fig 4A). Conversely, phosphoproteomics indicated fewer down-regulated phosphorylated peptides, showing an expression trend opposite to that observed in the proteomics results (Fig 4B).

### Molecular function analysis

**Fig 5:**
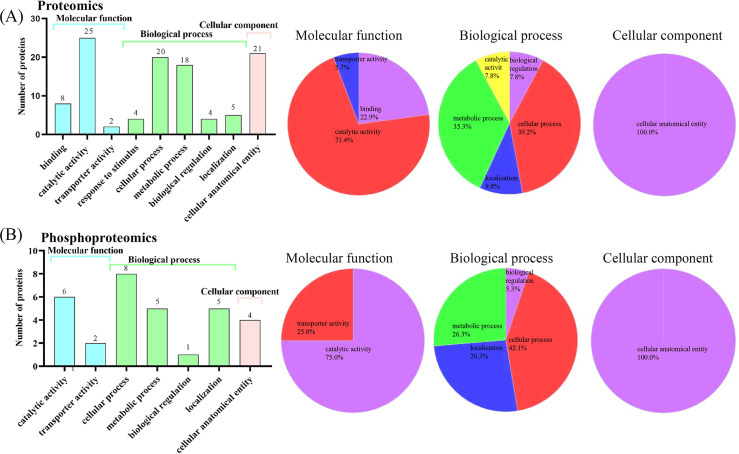
Gene ontology enrichment analysis of differentially expressed proteins in the ovary of female ticks under different treatments.

GO functional annotation analysis was performed on differentially expressed proteins in tick ovaries, categorizing them into three main areas: molecular functions, biological processes, and cellular components (Fig 5A). The results from DIA quantitative proteomics indicated that proteins enriched within the molecular functional category were predominantly involved in binding, catalytic activity, and transporter activity. Specifically, proteins associated with catalysis and binding accounted for 71.4% and 22.9% of the total protein count, respectively. In the phosphoproteomics analysis, proteins demonstrating catalytic and transporter protein activities accounted for 75% and 25% of the total proteins identified. This underscores the key role of enzymes and energy transfer-related proteins during toxin infection of tick ovaries. Within the biological processes category, the DIA quantitative proteomics analysis revealed that cellular processes and metabolic processes were the most abundant, accounting for 39.2% and 35.3% of the total proteins, respectively. Similarly, phosphoproteomics analysis showed that cellular processes, localization processes, and metabolic processes accounted for 42.1%, 26.3%, and 26.3% of the total proteins. These findings highlight the essential roles of cellular and metabolic processes in sustaining life activities and maintaining the dynamic balance of the internal environment in organisms. In the cellular components category, both DIA quantitative proteomics and Phosphoproteomics enriched for cellular anatomical entities (CEAs), such as nuclei, mitochondria, plastids, vesicles, vesicles, ribosomes, and the cytoskeleton. The enrichment of these cellular components suggests their fundamental role in the physiological functions of tick ovaries and its response to toxin challenges.

**Fig 6:**
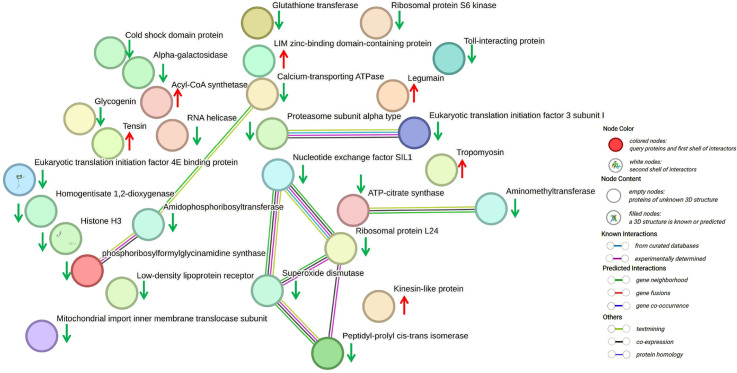
Protein interaction analysis of differentially expressed proteins. Red arrows represent upregulated proteins, while green arrows represent downregulated proteins. Circles indicate known interactions: light blue for curated database interactions; purple for experimentally determined interactions; green for gene neighborhood; red for gene fusions; and dark blue for gene co-occurrence. Edges represent protein-protein associations, which indicate that the proteins jointly contribute to a shared function; this does not necessarily imply physical binding.

**Fig 7:**
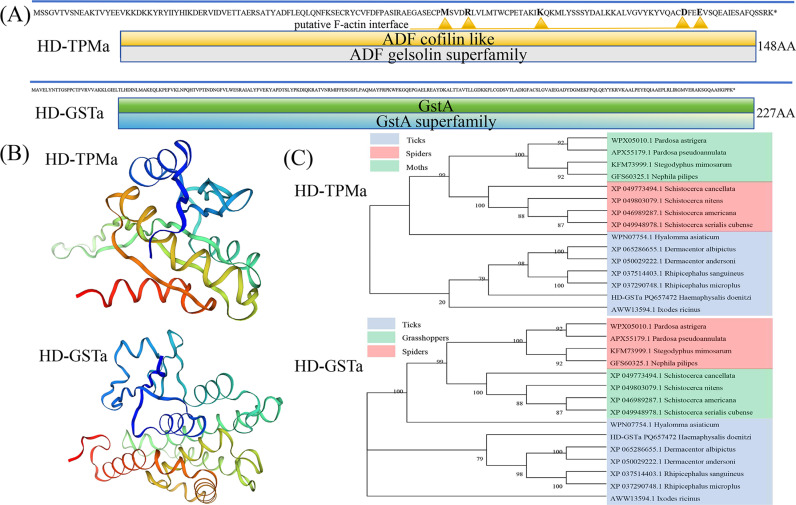
Bioinformatic analysis of two proteins characterized from *H. doenitzi.* (A) Schematic diagram of the conserved structure domains of proteins. (B) the predicted tertiary structures; (C) phylogenetic tree of the amino acid sequences.

To further investigate the biological functions of differentially expressed proteins in tick ovaries, KEGG pathway analyses were conducted on 860 differentially expressed proteins and 282 phosphorylated peptides. The DIA quantitative proteomics identified 31 significantly enriched proteins involved in multiple biological pathways (Table 2). These pathways encompass lysosome, regulation of the actin cytoskeleton, ribosome, RNA transport, citrate cycle (TCA cycle), oxidative phosphorylation, tyrosine metabolism, drug metabolism, glyoxylate and dicarboxylic acid metabolism, vitamin B6 metabolism, purine metabolism, protein processing in the endoplasmic reticulum, RNA degradation, and Ras signaling pathway. In contrast, phosphoproteomics identified 10 significantly enriched phosphorylated proteins involved in different signaling and metabolic pathways (Table 3). These pathways include longevity regulating pathway, protein digestion and absorption, PI3K-Akt signaling pathway, Hedgehog signaling pathway, glutamatergic synapse, mTOR signaling pathway, complement and coagulation cascades, and regulation of the actin cytoskeleton. The KEGG-string protein interaction analysis of all enriched differential proteins provided significant visualization of the differential status of these proteins (Fig 6). In this study, we genetically cloned and knocked down tropomyosin (HD-TPMa: PQ657471) and glutathione S-transferase (HD-GSTa: PQ657472) to investigate their key roles in the detoxification process of the tick ovary (Fig 7). Collectively, these findings reveal a potential role for differentially expressed proteins in modulating the response of ticks’ ovaries to external stimuli.

**Table 2:** Differentially expressed proteins in tick ovaries.

NO.	Accession	Description	Expression level	KEGG pathway
1	A0A9J6G6L2	Legumain	Up	Lysosome
2	A0A9J6G4H4	Alpha-galactosidase	Down	Lysosome
3	A5Z1D9	Tropomyosin	Up	Regulation of actin cytoskeleton
4	A0A9J6H2M6	60S ribosomal protein L18a	Down	Ribosome
5	E3UBG2	Ribosomal protein L24	Down	Ribosome
6	A0A9J6GQI9	RNA helicase	Down	RNA transport
7	A0A7S5D2J9	Eukaryotic translation initiation factor 3 subunit I	Down	RNA transport
8	A0A9J6GAK4	ATP-citrate synthase	Down	Citrate cycle (TCA cycle)
9	A0A9J6GC87	Dihydrolipoyllysine-residue succinyltransferase	Down	Citrate cycle (TCA cycle)
10	A0A9J6H3C4	Homogentisate 1,2-dioxygenase	Down	Tyrosine metabolism
11	A0A9J6GNM3	Glutathione S-transferase	Down	Drug metabolism
12	A0A9J6FJL4	Aminomethyltransferase	Down	Glyoxylate and dicarboxylate metabolism
13	A0A9J6G3B9	pyridoxal 5’-phosphate synthase	Down	Vitamin B6 metabolism
14	A0A9J6GEH5	phosphoribosylformylglycinamidine synthase	Down	Purine metabolism
15	A0A9J6FD15	inorganic diphosphatase	Down	Oxidative phosphorylation
16	A0A9J6G653	Cytochrome c oxidase subunit 5A, mitochondrial	Down	Oxidative phosphorylation
17	A0A9J6F9L3	Nucleotide exchange factor SIL1	Down	Protein processing in endoplasmic reticulum
18	A0A9J6GP01	Cold shock domain protein	Down	RNA degradation
19	A0A9J6GXE7	Ras-related protein Rac1	Down	Ras signaling pathway
20	A0A9J6HCQ7	Histone H3	Down	Signaling pathways regulating pluripotency of stem cells
21	A0A9J6GKD2	Acyl-CoA synthetase	Up	PPAR signaling pathway
22	A0A9J6GXI9	Toll-interacting protein	Down	Toll-like receptor signaling pathway
23	A0A9J6GG41	Calcium-transporting ATPase	Down	cAMP signaling pathway
24	A0A9J6FKU2	Proteasome subunit alpha type	Down	Proteasome
25	A0A9J6GDR0	Amidophosphoribosyltransferase	Down	Alanine, aspartate and glutamate metabolism
26	A0A9J6FFW8	Glycogenin	Down	Starch and sucrose metabolism
27	A0A9E8G7E3	Superoxide dismutase	Down	MAPK signaling pathway
28	A0A9J6H241	Peptidyl-prolyl cis-trans isomerase	Down	RIG-I-like receptor signaling pathway
29	A0A9J6GE24	Mitochondrial import inner membrane translocase subunit	Down	Phagosome
30	A0A9J6H8X9	Low-density lipoprotein receptor	Down	Thyroid hormone synthesis
31	A0A9J6FFZ4	Pre-mRNA-processing factor 19	Down	Ubiquitin mediated proteolysis

**Table 3:** Differentially expressed proteins with phosphorylation modifications in tick ovaries.

NO.	Accession	Description	Phosphopeptide	Expression level	KEGG pathway
1	A0A9J6H817	LIM zinc-binding domain-containing protein	LLQR	Up	Longevity regulating pathway
2	A0A9J6G8J0	Tensin	PIIPSR	Up	Protein digestion and absorption
3	A0A2D1C0A7	Heat shock protein 83	DDEEEEEK	Up	PI3K-Akt signaling pathway
4	A0A9J6FQW2	Kinesin-like protein	PLVAGGR	Up	Hedgehog signaling pathway
5	A0A9J6FEW0	Guanylate-kinase-associated protein	PMSSER	Up	Glutamatergic synapse
6	I7GSF0	Eukaryotic translation initiation factor 4E binding protein	PEHKVTTSR	Down	mTOR signaling pathway
7	A0A9J6GTW3	Ribosomal protein S6 kinase	PAIPTR	Down	mTOR signaling pathway
8	A0A9J6FT92	BAG domain-containing protein	ADHAAAGEPK	Down	Complement and coagulation cascades
9	A0A9J6FMS1	Moesin/ezrin/radixin homolog 1	PGVQPPGAK; HGGKELPGAVK	Down	Regulation of actin cytoskeleton
10	A0A9J6FBQ6	K Homology domain-containing protein	PDAK	Down	Regulation of actin cytoskeleton

### Enzyme activity

**Fig 8:**
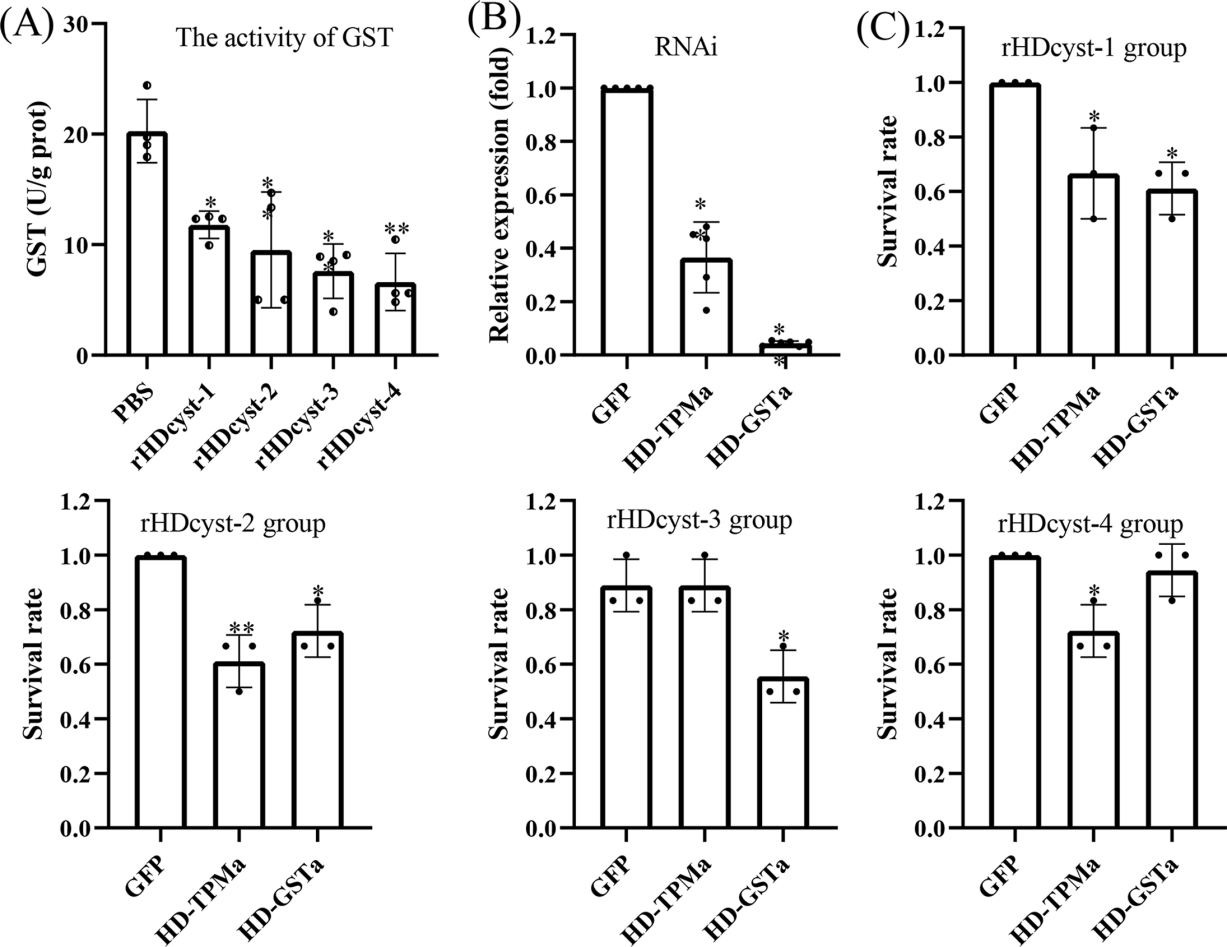
(A) Effects of cystatin antibodies on GST of unfed female adult *H. doenitzi*. (B) RNA interference efficiency of two genes in *H. doenitzi* (GFP, green fluorescent protein). (C) Ticks survival rate at different cystatin antibodies after RNA interference. *P < 0.05, **P < 0.01 indicates statistically significant differences compared with the control group.

The effect of rabbit-derived cystatin antibodies on glutathione S-transferase (GST) activity in unfed adult female ticks was assessed. The GST activity in the control group was found to be 1.72-fold, 2.13-fold, 2.67-fold, and 3.06-fold higher than that in the experimental group inoculated with rHDcyst-1, rHDcyst-2, rHDcyst-3, and rHDcyst-4 antibodies, respectively (Fig 8A). This finding reveals that GST detoxification enzyme activity undergoes a significant reduction following cystatin antibody treatment.

### RNAi

RNAi successfully reduced the relative expression of HD-TPMa and HD-GSTa by 63.4% and 95.6%, respectively (Fig 8B). After 24 h of knockdown treatment, we performed an antibody immersion test on ticks, resulting in a significant increase in tick mortality after 72 h (Fig 8C). The mortality of ticks with knocked-down HD-TPMa and HD-GSTa ticks was significantly increased following treatment with rHDcyst-1 and rHDcyst-2 antibodies. In addition, a significant rise in HD-GSTa mortality was observed under rHDcyst-3 antibody immersion, while rHDcyst-4 antibody immersion led to a significant increase in HD-TPMa mortality after knockdown. These results indicate a significant increase in tick mortality by targeting the knockdown of the HD-TPMa and HD-GSTa target genes, confirming the central role of these two proteins in tick ovarian reproductive development.

## Discussion

In this study, we investigated the effects of cystatin antibody toxins on tick ovaries, focusing on the potential causes of ovarian cell damage and decreased egg production [1,2]. To unravel the molecular mechanisms underlying ovarian detoxification, we conducted comprehensive HE staining, immunohistochemical, and immunofluorescence analyses of toxin-stressed ovarian tissues. The molecular response of the ovary was examined through proteomics and phosphoproteomics. Proteomics identified crossover proteins among 860 proteins, with 31 showing significant differentiation in biological pathways. Phosphoproteomics revealed significant phosphorylation sites in 43 proteins, 10 of which demonstrated significant changes in their pathway. Key pathways involved in ovarian detoxification included ribosomes, regulation of the actin cytoskeleton, RNA transport, the citric acid cycle, drug metabolism, and the mTOR signaling pathway. Proteins involved in detoxification within the key pathways of actin cytoskeleton regulation and drug metabolism have been extensively studied. We cloned the immunogenic HD-TPMa and HD-GSTa, which are implicated in detoxification, and verified the role of GST in toxin-infested ovaries through enzyme activity tests. The effects of HD-TPMa and HD-GSTa on the survival of *H. doenitzi* under cystatin antibody toxins were explored using RNAi. The increased tick mortality confirmed that both proteins regulate the physiological processes in response to toxins. This study provides new molecular targets for tick control, which could help reduce the risk of tick-borne diseases and improve public health by exploring the tick ovary’s stress response to toxins.

The reduction in the number of toxin-infested oocytes was evident in ticks whose ovaries had been subjected to cystatin antibody toxin stress. HE staining revealed ruptured oocytes, providing indirect evidence that the toxin adversely affects ovarian function. Immunohistochemical analysis revealed the distribution of cystatin antibodies in the ovaries, showing their presence in both control and toxin-infested groups, suggesting that cystatin plays an important role in the immune response of the ovary. Immunofluorescence analysis showed that cystatin antibodies exhibited a bright positive fluorescent reaction in toxin-infested ovaries, indicating an active ovarian defense against the toxin [12,13]. These results highlight potential defense strategies employed by tick ovaries when facing cystatin antibody toxin stress. Future studies should explore the use of vaccination of livestock for tick control in combination with pyrethroid compounds [2]. Cystatin in ticks is expressed in different tissues and inhibits cathepsins, indicating that immunocontrol using cystatin antibodies has potential for a variety of tick species [1]. However, key issues such as the stability and efficacy of the antibody must be addressed. Further studies are needed to assess its stability under different environmental conditions and to optimize its delivery and action in ticks. In addition, cystatin antibodies must be evaluated for their impact on non-target organisms and ecosystems before application, as ensuring the safety of this control approach is critical for public health.

To investigate the molecular mechanisms of the toxin on tick ovaries, proteomics, and phosphoproteomics analyses were performed. Proteomics identified 31 proteins involved in various pathways, while phosphorylation proteomics identified 10 proteins. The main pathways include lysosome, regulation of the actin cytoskeleton, ribosome, RNA transport, citrate cycle, and drug metabolism. In contrast, the pathways enriched in phosphorylation were the longevity regulating pathway, protein digestion and absorption, PI3K-Akt signaling pathway, hedgehog signaling pathway, glutamatergic synapse, mTOR signaling pathway, complement and coagulation cascades, regulation of the actin cytoskeleton.

Notably, there was high expression of legumain and low expression of Alpha-galactosidase (alpha-Gal) in the lysosomal pathway. Legumain is recognized as a tick midgut digestive protease [14]. The deletion of specific Legumain in Haemaphysalis longicornis resulted in midgut damage, and knockdown of the Legumain gene led to a significant delay in the onset of oviposition and a reduction in the number of eggs [15]. The increase of legumain protein indicates that the ovary regulates normal development. Additionally, the expression of cathepsins also increases rapidly after scorpion venom ingestion [16], while tick cells elevate alpha-Gal levels following *Anaplasma phagocytophilum* infection to control bacterial infections [17]. Alpha-Gal in *Amblyomma americanum* is involved in autophagic responses and galactose metabolism [18]. Autophagy functions as an evolutionarily conserved degradation system for eliminating intracellular components via the lysosomal pathway [19]. In response to toxin infestation in the ovary of *H. doenitzi*, lysosomes may defend against damage through autophagy, and potential regulation of cytoskeleton rearrangement and apoptotic processes was observed during *Amblyomma americanum* attacks on *Escherichia coli* [20].

Furthermore, tropomyosin protein was found to be highly expressed in the regulation of actin cytoskeleton following the tick ovaries’ response to cystatin antibody attack, while phosphorylation modifications of Moesin/ezrin/radixin homolog 1 and K Homology domain-containing protein were attenuated. Tropomyosin may play a key role in the regulation of cell function by enhancing the stability of actin microfilaments [21]. High expression levels of venom protease and tropomyosin were also noted in the female parasitic wasps *Lysiphlebia japonica* Ashmead [22]. Proteins from the ezrin, radixin, and moesin (ERM) family are known to regulate cell and tissue morphogenesis, and the activation of moesin that inhibits STRIPAK phosphatase activity can lead to defective cell morphology during mitosis and compromise epithelial tissue integrity [23]. Additionally, K Homology domain-containing protein may function as a scaffolding protein interacting with *Drosophila* dynamin [24].

Low expression of 60S ribosomal protein L18a and Ribosomal protein L24 was observed in the ribosomal pathway. In *Aedes aegypti*, RNAi-mediated knockdown of the 60S acidic ribosomal protein P1 has been shown to prevent egg development in adult female yellow fever mosquitoes [25]. Additionally, natural peptide antigens in the Plasmodium 60S ribosomal protein L6 (RPL6) confer cell-mediated immunity to malaria in liver TRM cell mice [26]. Sublethal concentrations of Cry1Ab treatment in *Spodoptera litura* down-regulate the mRNA expression of detoxification genes involved in the ribosomal pathway [27]. Moreover, *Pseudomonas aeruginosa* employs a virulence mechanism that induces ribosomal degradation of the host ribosome, impairing host translation and blocking the antimicrobial response [28]. It is hypothesized that cystatin antibody toxins harm ovarian ribosomes, thereby reducing egg production [29].

In the RNA transporter pathway, low expression of RNA helicase and eukaryotic translation initiation factor 3 subunit I (eIF3f1)was noted. RNA helicases play a role in the innate immune response, with three RNA deconjugases in *Laodelphax striatellus* being highly expressed in the ovary; knockdown of these RNA deconjugase genes disrupts ovarian and oocyte development [30]. Among these, RNA helicase Dhx15 regulates CHIKV arbovirus replication by controlling glycolysis in mosquito cells [31]. In mammals, eIF3 is a multisubunit complex [32], and eIF3f1 is essential for antimicrobial innate immune defense in *Drosophila melanogaster* [33]. Furthermore, knockdown of eIF3m inhibited *Rhodnius prolixus* molting, leading to premature mortality and impairing adult female ovary development and egg laying [34].

ATP-citrate synthase and Dihydrolipoyllysine-residue succinyltransferase (DLST) were found to be down-regulated in the citrate cycle pathway. Reducing the activity of ATP-citrate synthase may contribute to the regulation of individual lifespan in *Drosophila* [35]. Lower levels of ATP-citrate synthase impair mitochondrial energy production [36], while impaired function of DLST, a component of α-ketoglutarate dehydrogenase complex (KGDHC), exacerbates mitochondrial ATP deficiency [37]. DLS plays a key role in nutrient transformation [38]. The low expression of ATP-citrate synthase and DLST leads to decreased energy metabolism, which may facilitate toxin invasion [39]. Given that cystatin antibody toxin is a foreign substance, ingestion of the drug metabolic pathway by ticks alters glutathione S-transferase (GST) activity. GST is a multifunctional detoxification enzyme, and it typically increases with insecticide treatment [40]. Enhanced GST detoxification enzyme activity is essential for safeguarding individual longevity mechanisms [41]. GST is also highly expressed in the ovaries of *Locusta migratoria manilensis*, where it helps maintain ovarian development and reproduction against pyrethroids [42].

Phosphoproteomics revealed that the eukaryotic translation initiation factor 4E binding protein (eIF4E) and ribosomal protein S6 showed low expression levels in the mTOR signaling pathway kinase, particularly regarding S6 kinase (S6K). eIF4E controls a key step in translation initiation, and its phosphorylation promotes normal development of reproductive organs [43]. In *Drosophila melanogaster*, eIF4E regulates non-apoptotic caspase activity during individuation and is involved in cellular autophagy [44]. S6 kinase (S6K) is an important mediator in regulating cell growth and proliferation at the translational level [45]. *Drosophila* lacking the S6 kinase gene (dS6K) exhibit extreme developmental delays and severe size reduction [46]. The decrease in phosphorylation levels of both eIF4E and S6K suggests that cystatin antibody toxins may effectively infest tick ovaries.

The phosphorylation of LIM zinc-binding domain-containing protein (LIM) in the longevity regulating pathway is enhanced. Conserved cysteine and histidine residues in the LIM structural domain indicate metal-binding roles, which have been implicated in muscle differentiation in vertebrates [47]. The peak expression of LIM gene expression occurs during late embryogenesis, and these proteins can bind to the actin cytoskeleton [48]. Tensin phosphorylation is also enhanced in the protein digestion and absorption pathway. Tensin is associated with connecting integrins to the cytoskeleton and various signaling pathways [49]. Damaged mitochondria are eliminated through selective mitochondrial autophagy, a mechanism requiring tensin factor phosphorylation [50]. Furthermore, enhanced phosphorylation of heat shock protein 83 has been observed in the PI3K-Akt signaling pathway. The heat shock protein Hsp90 is known to associate with various cell signaling proteins [51], while Hsp70, another family member, helps clear viral infections [52]. In the Hedgehog signaling pathway, Kinesin-like protein phosphorylation is enhanced [53]. Kinesin-like proteins are crucial for organizing contractile actin at the equator of dividing cells [54]. Conversely, phosphorylation of guanylate-kinase-associated protein is diminished in the Glutamatergic synapse, which may indicate that ticks experience irritant pain [55]. This protein is involved in the indirect proliferation control of *Drosophila* epithelial cells [56]. Overall, toxin infestation appears to damage ovaries and reduce tick injury.

The regulation of ovarian responses to toxins is extremely complex. The low expression of HD-GSTa in toxin-infested ovaries suggests that the toxin may impair tick detoxification by inhibiting GST activity. GST plays a key role in the detoxification of numerous exogenous substances [57]. Previous studies have shown that GST exhibits high enzymatic activity in insects in response to toxin stresses, such as insecticides [58]. For example, LsGSTE1 in *Lasioderma serricorne* metabolizes pesticides and prevents lambda-cyhalothrin (LCT) induced oxidative stress [59]. Similarly, overexpression of *BdGSTd5* in *Bactrocera dorsalis* contributes to the detoxification of malathion [60]. This study primarily established a correlation between cystatin antibody exposure and tick ovarian dysfunction. The decrease in GST enzyme activity observed with cystatin antibodies may be linked to the disruption of detoxification pathways. For example, in drug-resistant insects, GST expression is significantly up-regulated in response to insecticides [61]. Additionally, tropomyosin, which is primarily associated with cytoskeletal stability, may indirectly affect detoxification pathways through cellular stress responses [62].

To verify the role of toxins in the detoxification process, HD-GSTa and HD-TPMa were genetically cloned. HD-GSTa consists of 227 amino acids in length and contains the GSTA structural domain, showing greater homology to tick proteins. HD-TPMa, on the other hand, is 148 amino acids long and features the ADF gelsolin superfamily structure, which is more conserved in ticks compared to spiders and moths. After identifying these genes, RNAi knockdown experiments were performed, followed by treatment of the surviving ticks with cystatin antibody toxins. The mortality rate of ticks after the knockdown of HD-GSTa was significantly higher in groups treated with rHDcyst-1, rHDcyst-2, and rHDcyst-3. Similar findings were observed when Slgste1 expression was inhibited in *Spodoptera litura*, leading to reduced larval growth and feeding rates [63]. Insecticide bioassays revealed increased mortality in *Diaphorina citri* after RNAi knockdown of DcGSTe2 and DcGSTd1 [64]. These results suggest that HD-GSTa in the ovary plays a unique role in the detoxification of cystatin antibody toxins. Additionally, knockdown of HD-TPMa significantly increased tick mortality in the rHDcyst-1, rHDcyst-2, and rHDcyst-4 treatment groups. Previous studies have shown that knockdown of tropomyosin in *Periplaneta americana* results in failed ovulation and affects reproductive development [65]. Likewise, knockdown of DcTm1-X1 in *Diaphorina citri* significantly increased mortality rates and the infection rate of CLas pathogen at various time points [66]. The present study demonstrates that both HD-GSTa and HD-TPMa in the ovary play essential roles in the detoxification of cystatin antibody toxins.

The limitations of this study include the lack of quantitative data on ovarian morphology. The RNAi results showed an effect on tick survival, but a direct link to ovarian dysfunction is unknown. These findings have important implications for tick and tick-borne disease control and public health. Future research should focus on quantitative analysis of ovarian morphology, elucidation of the molecular mechanisms underlying RNAi effects on tick survival and ovarian function, validation of these effects in host animals through *in vivo* experiments, exploration of cross-species applications, development of cystatin-based vaccines and therapeutics, and integration of omics technologies to identify additional targets and pathways [67]. Working in these directions will improve our understanding of tick biology and contribute to more effective tick control strategies.

## Conclusions

This study primarily established a correlation between cystatin antibody exposure and tick ovarian dysfunction. It confirms that cystatin antibodies interfere with ovarian development and emphasizes the similarity of their response to insect infection. The significant down-regulation of GST enzyme activity after toxin treatment underscores the molecular mechanisms involved in the detoxification process in the ovary. The successful cloning and knockdown of HD-GSTa and HD-TPMa reveal their crucial roles in tick detoxification metabolism, contributing to our understanding of detoxification functions in tick ovaries and providing an important scientific basis for pest control strategies.

## Supporting information

S1 Fig Quantitative analysis of the fluorescence intensity of ovarian-specific antibodies. Different letters indicate statistical differences (*P* < 0.05).
(TIF)
