# Supplementary figures and images for: Cystatin antibodies interfere with ovary development in Haemaphysalis doenitzi (Acari: Ixodidae)
Source: PLoS Negl Trop Dis. 2025 May 7;19(5):e0013064. doi: 10.1371/journal.pntd.0013064 (PMC12057904; doi:10.1371/journal.pntd.0013064)

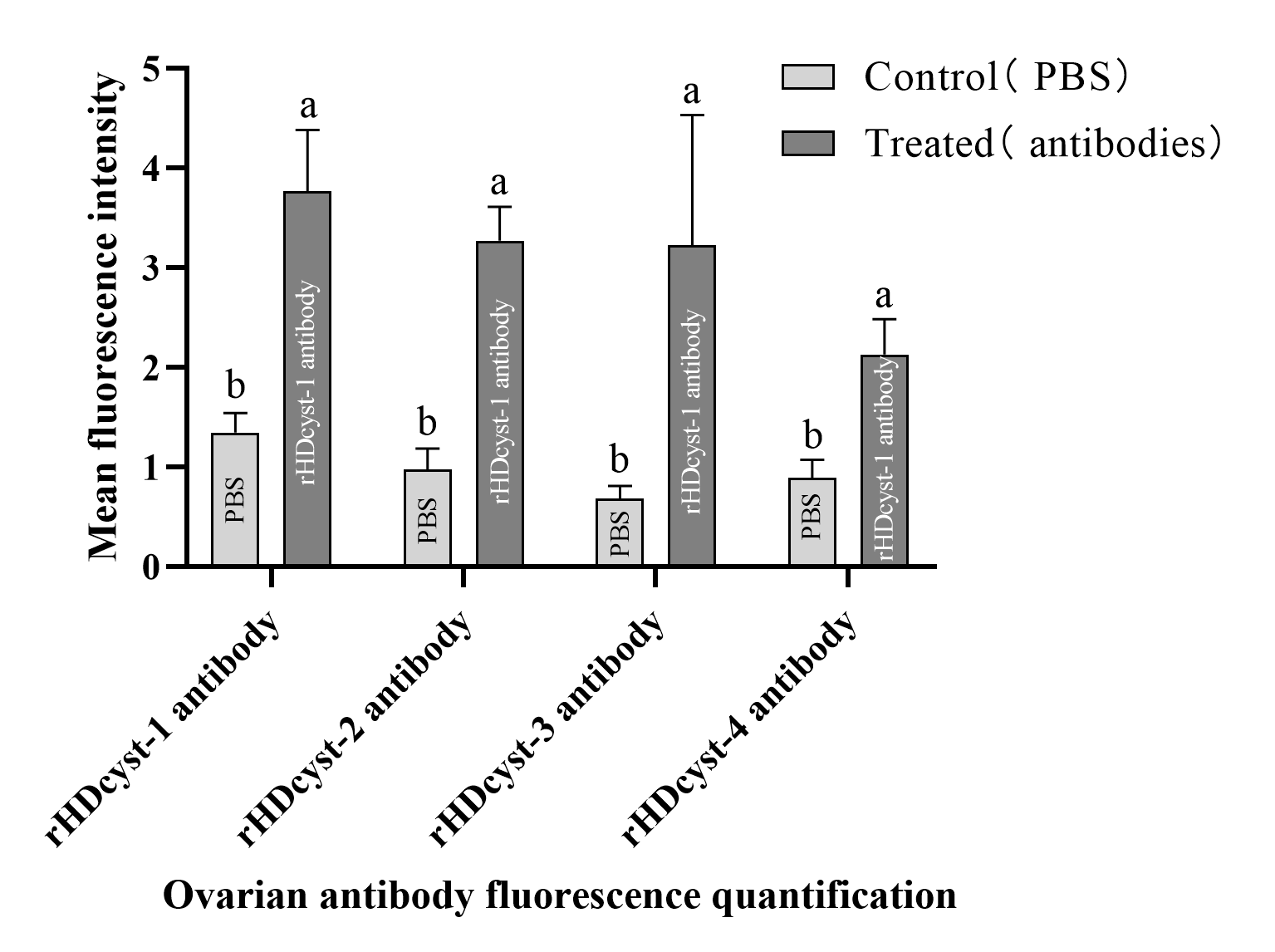

Supplement: S1 Fig — Different letters indicate statistical differences (P < 0.05). (TIF) [file pntd.0013064.s001.tif]
